# High-Grade Vaginal Intraepithelial Neoplasia: Clinical Profile, HPV Vaccination Status, and Treatment Outcomes at Two Italian Referral Centers

**DOI:** 10.3390/vaccines13111089

**Published:** 2025-10-24

**Authors:** Niccolò Gallio, Mario Preti, Renzo Boldorini, Cristina Cavagnetto, Fulvio Borella, Federica Bevilacqua, Ilaria Barbierato, Raffaella Ribaldone, Enrica Bovio, Chiara Airoldi, Valentino Remorgida, Luca Marozio, Alberto Revelli

**Affiliations:** 1 Gynecology and Obstetrics 2U, “AOU Città della Salute e della Scienza di Torino”, Department of Surgical Sciences, University of Turin, 10126 Turin, Italy; alberto.revelli@unito.it; 2 Gynecology and Obstetrics 1U, “AOU Città della Salute e della Scienza di Torino”, Department of Surgical Sciences, University of Turin, 10126 Turin, Italy; mario.preti@unito.it (M.P.); fulvio.borella87@gmail.com (F.B.); ila.barbie@hotmail.it (I.B.); luca.marozio@unito.it (L.M.); 3 Department of Health Sciences, University of Eastern Piedmont, 28100 Novara, Italy; renzo.boldorini@med.uniupo.it (R.B.); raffaellarib@gmail.com (R.R.); enrica.bovio@gmail.com (E.B.); valentino.remorgida@med.uniupo.it (V.R.); 4 Department of Maternal-Neonatal and Infant Health, Division of Obstetrics and Gynaecology, Ospedale Degli Infermi, University of Turin, 13900 Biella, Italy; cristinacavagnetto@yahoo.it; 5 Gynecology and Obstetrics 3U, “AOU Città della Salute e della Scienza di Torino”, Department of Surgical Sciences, University of Turin, 10126 Turin, Italy; fede.bevi23@gmail.com; 6 Department of Translational Medicine, University of Eastern Piedmont, 28100 Novara, Italy; chiara.airoldi@uniupo.it

**Keywords:** HPV, vaginal intraepithelial neoplasia, vagina, anogenital cancer, HSIL

## Abstract

**Background**: There is limited available data about the natural history and clinical characteristics of patients affected by high-grade vaginal intraepithelial neoplasia (or vaginal intraepithelial neoplasia 2/3 or vaginal high-grade squamous intraepithelial lesion, HSIL). The aim of the study was to review clinical characteristics and treatment outcomes of a large cohort of patients with vaginal HSIL. **Methods**: A retrospective analysis was performed for patients with histologically confirmed VaIN 2/3 treated at two Italian referral centers from 2003 to 2022. Demographics, referring cytology, associated cervical and vulvar HSIL treatment modalities, follow-up visits, and vaccination status were recorded. The primary outcome was risk of recurrence; the secondary outcome was risk of progression to invasive carcinoma after treatment. **Results**: 323 women were included in the analysis: 69.7% (225) had VaIN2, and 30.3% (98) had VaIN3. Mean age at diagnosis was 55.43 years (range 24–93 years). 20.4% had a previous hysterectomy, mainly for cervical intraepithelial neoplasia (CIN)/invasive squamous carcinoma (64.6%). In total, 55.2% underwent ablative therapy, and 44.8% underwent excisional treatment. Over a mean follow-up of 62.43 months, 22.0% of patients recurred as vaginal HSIL. At univariate analysis older age, VaIN grade 3, previous hysterectomy, associated cervical lesions, associated vulvar HSIL, and excisional treatment were significantly associated with increased risk of recurrence. At multivariate analysis, only hysterectomy and excisional treatment maintained significance. Five patients progressed to invasive vaginal carcinoma, with a median time to invasion of 87.1 months. **Conclusions**: The risk of recurrence of vaginal HSIL is higher in patients treated with excisional methods and/or those who have undergone hysterectomy for both benign and preinvasive lesions. Long-term follow-up is essential to monitor clinical outcomes and prevent disease progression.

## 1. Introduction

Vaginal intraepithelial neoplasia (VaIN) is a rare condition that precedes invasive squamous vaginal carcinoma, with an incidence rate of 0.2–0.3 cases per 100,000 women in the US [1]. VaIN is closely associated with persistent infection by high-risk human papillomavirus (HPV) genotypes, particularly HPV 16, followed by other genotypes such as 58, 73, and 31 [2,3,4]. Other risk factors include immunosuppression, cigarette smoking, and a history of other gynecological malignancies [5].

VaIN is usually asymptomatic until it progresses to invasive cancer. Due to the lack of specific symptoms, Pap smears and HPV testing are crucial for early detection of VaIN [6]. Indeed, VaIN might be detected during cervical cancer screening, with an abnormal pap test in the context of a normal cervical colposcopy.

VaIN is histologically classified into three grades based on the affected thickness of the vaginal epithelium by abnormal cells: VaIN 1 (lower third), VaIN 2 (lower two-thirds), and VaIN 3 (full thickness) [7]. According to 2012 LAST terminology, low-grade dysplasia (low-grade intraepithelial lesion, LSIL) corresponds to VaIN1, while high-grade dysplasia (high-grade intraepithelial lesion, HSIL) encompasses VaIN2 and VaIN3 [7].

High-grade VaIN may in some cases progress into invasive vaginal cancer. The risk of cancer ranges from 2 to 12% [1,8,9], and this risk increases with the duration of follow-up. There is a possibility that vaginal cancer may have been undetected at the time of initial VaIN diagnosis: the risk of occult cancer is reported to be 4.6%, and it can be as high as 10.3% in the VaIN3 cohort [10].

HPV vaccination is a critical primary prevention intervention, and it has proven its efficacy against cervical HPV infection, high-grade preinvasive lesions, and invasive cancers, especially when administered at an early age before sexual debut [11]. It is reasonable to expect that the effectiveness of prophylactic HPV vaccination will extend to the prevention of non-cervical HPV-associated malignancies, with the most pronounced benefits observed in women who are vaccinated at an early age, since more than 70% of VaIN are associated with HPV types included in the 9-valent vaccine [3]. However, since the median age at VaIN diagnosis is postmenopausal, an adjuvant vaccination post-treatment is anticipated to be of small benefit in order to prevent recurrences. Recent cohort studies from Denmark have shown a significant decline in the incidence of VaIN among women under 30 years of age following the introduction of the HPV vaccine in 2006, with an annual reduction of nearly 16% [12]. In women aged 30–39 years, a modest but statistically non-significant decrease was also observed. Additionally, another Danish study reported lower rates of vaginal high-grade squamous intraepithelial lesions (HSIL) among vaccinated women compared to their unvaccinated counterparts, with an adjusted hazard ratio of 0.3 (95% CI: 0.13–0.68) [13]. The protective effect was even more pronounced among those vaccinated before the age of 16 years, where the adjusted hazard ratio was 0.16 (95% CI: 0.04–0.55) [13].

VaIN management depends on the grade and extent of the lesions. Low-grade lesions may resolve spontaneously or be approached with close monitoring, while high-grade lesions require interventions in most cases [6].

Treatment options for VaIN include topical therapies, ablation either with CO_2_ laser vaporization or radiofrequency, and surgical excision [14]. Topical agents like imiquimod can be applied directly to the affected area [6,15]. Laser ablation uses focused energy to target abnormal tissue [16], while surgical excision involves the removal of the affected part of the vagina, which may be performed using a laser, radiofrequency scalpel, or cold knife. In some cases, a combination of these treatments may be used to achieve optimal results [17].

Regular follow-up is crucial, as recurrence is reported in a wide range from 2 to 50% according to various series [18], and long-term surveillance is necessary to monitor for any sign of progression or persistence. Additionally, vaccination against high-risk HPV types can play a preventive role in reducing the VaIN recurrences [12].

The goal of this study is to describe the clinical characteristics of a large group of patients with HG (high-grade) VaIN, exploring risk factors for recurrence and HPV vaccination status. This study represents the second part of a multicentric study where HPV type at diagnosis and epidemiological characteristics of the patients were reported [3].

## 2. Materials and Methods

We performed a retrospective analysis of all women with a histopathological diagnosis of VaIN 2/3 at the Department of Surgical Sciences, Sant’Anna Hospital, University of Torino, Torino, Italy, and at the Ospedale Maggiore della Carità, Novara, Italy, from 2003 to 2022. The following clinical and pathological data were retrieved from medical charts: age at diagnosis, presence of concomitant or previous histologically proven cervical or vulvar squamous intraepithelial lesions (cervical HSIL or vulvar HSIL), presence of immunodepression (either iatrogenic or HIV-related), clinical reason for hysterectomy if performed, parity, site of VaIN, type of treatment, and vaccination status with linkage with public vaccination policy databases.

Topical vaginal estrogen therapy was prescribed preoperatively to postmenopausal women. Patients underwent general or local anesthesia, depending on the site of the lesions and comorbidities. A preoperative colposcopy was performed with application of 5% acetic acid and Lugol’s solution to guide treatment. Excisional procedures included either cold knife, radiofrequency, or CO_2_ laser excision. A specialized gynecologist performed a colposcopy-guided hand-directed laser excision. The spot size was focused at 0.2 mm, an output between 6 and 10 watts, and a focal spot at 25 to 30 cm. The ablative procedure involved laser vaporization, with a power density of 1130 W/mL over a 1.5 mm spot diameter to a depth of 1 mm, with at least 5 mm of safety margin, at 5 to 8 W.

Follow-up was scheduled every 6 months for the first two years and consisted of HPV and cytology testing, as well as colposcopy with directed biopsy if needed. Recurrence was defined as any histologically proven VaIN 2/3 on biopsy. All colposcopies were performed by gynecologists with expertise in the diagnosis and treatment of lower genital tract diseases.

Statistical analysis was performed using IBM SPSS^©^ statistical software (version 28.0). Descriptive statistics were reported as mean, median, standard deviation (SD), range, and interquartile range (IQR) for continuous variables and as absolute frequency and percentage for categorical variables. To test the difference between the two groups, the Wilcoxon–Mann–Whitney test and Fisher’s exact test were applied to continuous and categorical variables, respectively. Univariate and multivariate Cox proportional hazard models were performed to evaluate the association of recurrence with selected clinical parameters. The statistical significance threshold was *p* < 0.05.

The study was approved by the Research Ethics Committee for Human Biospecimen Utilization (Department of Medical Sciences—ChBU) of the University of Turin (n°2/2022, date of approval 22 February 2022). All patients included in our retrospective study were treated according to the ethical standards of our local committee on human experimentation and with the Helsinki Declaration and signed informed consent for the anonymous use of clinical and instrumental data for research purposes at the time of diagnosis.

## 3. Results

A total of 323 patients with biopsy-proven VaIN2/3 in the study period were included. The baseline characteristics of the population are detailed in Table 1. The mean age of the study cohort was 55.43 years, with VaIN3 patients significantly older than VaIN2 patients (52.74 vs. 61.59, *p* < 0.0001). Among the study cohort, 117 (36.56%) were nulliparous, while 203 (63.44%) had one or more previous pregnancies. Nulliparity was significantly more frequent in VaIN2 (83.76%) compared with VaIN3 patients (16.24%) (*p* < 0.0001). 24 (7.57%) presented with concurrent immunodepression (either iatrogenic or HIV-related). Of these, 17 (70.83%) were VaIN2 and 7 (29.17%) were VaIN3, with no statistically significant difference between groups (*p* = 0.9289).

A total of 66 patients (20.43%) of our cohort had a previous hysterectomy; 42 of them had it for cervical HSIL (*n* = 24) or invasive cervical cancer (*n* = 18). The mean age at the time of hysterectomy was 55.25 years (range 45–76).

The median interval between hysterectomy and VaIN diagnosis was 42.4 months (Q1; Q3: 17.56; 145 months). The time from hysterectomy and VaIN diagnosis for patients with CIN/cervical neoplasia indication was 30.5 months, while for those with benign lesions was 63 months (*p* < 000.1). Also, the time from hysterectomy to VaIN3 diagnosis was significantly lower compared to VaIN2 diagnosis, 32.23 vs. 62.10 months, *p* = 0.0164.

One patient in our cohort received primary HPV vaccination with the bivalent HPV vaccine. Six patients underwent HPV vaccination before VaIN treatment due to other indications, and 42 were vaccinated adjuvantly after treatment. However, univariate and multivariate analyses to assess the impact of vaccination on recurrence risk could not be performed due to the heterogeneity of available data—in particular, the wide variability in the timing between treatment and vaccination. Moreover, the structure of our cohort presents intrinsic limitations: follow-up began in 2002, while HPV vaccination programs were only introduced in Italy for 11-year-old girls in 2010, and adjuvant vaccination after treatment only became part of clinical practice in more recent years (2020). As a result, the number of patients with complete and comparable vaccination data is limited.

Initial therapy consisted of ablative treatment in 55.2% and excisional in 44.8%, with excisional therapy being more frequent in VaIN2 patients (57.04%) than in VaIN 3 (42.96%).

### 3.1. Follow-Up

**Figure 1:**
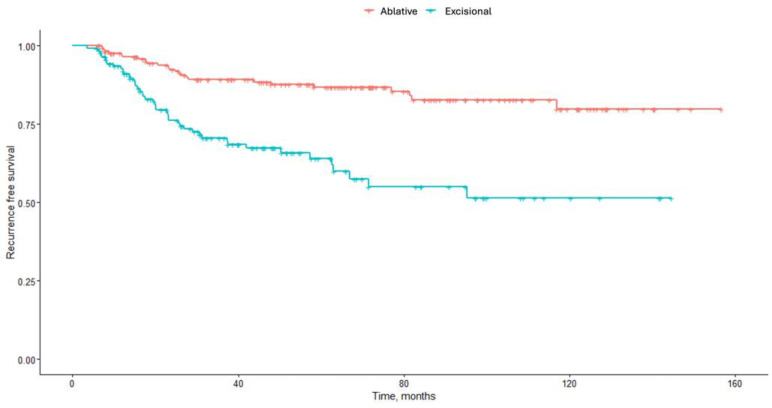
Recurrence-free survival after treatment (in red, ablative treatment; in blue, excisional treatment).

During a median follow-up of 62.43 months (Q1; Q3: 52.35-71.41 months), 22.0% of patients experienced recurrence (Figure 1). The 5-year cumulative recurrence risk was 25.8%, and the 10-year cumulative recurrence risk was 37.5%. The overall recurrence rates stratified for type of therapy were 13.7% for ablative treatment and 32.4% for excisional therapy (*p* < 0.001). 5-year recurrence cumulative risk was 12.5% for ablative treatment and 37.5% for excisional treatment, while 10-year recurrence cumulative risk was 19.3% for ablative treatment and 52.3% for excisional treatment. In particular, VaIN3 had a higher rate of recurrence compared to VaIN 2, 29.6% vs. 18.7%, respectively (*p* < 0.001).

A concurrent, previous, or subsequent cervical lesion was diagnosed in 34.67% of patients. In most cases (44.77%), a cervical lesion was concurrent with VaIN, while in 30.22% it preceded VaIN diagnosis. In 14.17% of patients, a cervical lesion was found during VaIN follow-up. Regarding the type of cervical lesion, a CIN3 was present in 37.50%, a CIN2 in 33.04%, and a CIN1 in 27.68%, while an invasive squamous carcinoma was present in 8.04% and adenocarcinoma in 1.79%.

A vulvar high-grade intraepithelial lesion was diagnosed in 17 women (5.26%). In half of these cases the lesion was synchronous with the VaIN diagnosis and, in the other half, was found during follow-up.

Regarding risk factors related to VaIN recurrence (Table 2), at univariate analysis older age, VaIN3, previous hysterectomy, associated cervical lesions, associated VHSIL, and excisional treatment were significant. At multivariate analysis, hysterectomy and excisional treatment maintained significance.

### 3.2. Risk of Vaginal Cancer

During follow-up, vaginal cancer developed in five patients (1.55%). Their characteristics are summarized in Table 3. The mean age of these patients was 57 years (range 43–72 years), which was not statistically different from other patients.

The progression rate was 0.44% and 4.08% for patients diagnosed with VaIN2 and VaIN3, respectively (*p* < 0.0001).

The mean time to invasion was 87.1 months after treatment (range 15.75–155.58 months). Four of the five patients (80%) were initially diagnosed with VaIN3 and only one with VaIN2. All patients who developed invasive cancer experienced a VaIN recurrence in a median time to recurrence of 11.75 months. Three patients underwent excisional treatment, and two received ablative treatment as their first treatment. All underwent repeated treatment before progression.

## 4. Discussion

Limited data are available in the literature regarding high-grade VaIN because of its rarity and difficulties in its diagnosis and treatment. While VaIN and CIN are closely linked, with a significant percentage of VaIN cases preceded or accompanied by a CIN diagnosis (34.67%), VaIN has a 100 times reduced incidence, despite sharing the same risk factors [19]. This difference in incidence reflects both biological and epidemiological factors: the cervix contains a transformation zone particularly susceptible to persistent high-risk HPV infection, whereas the vaginal epithelium is less prone to such changes. Routine cervical screening programs also detect CIN at higher rates, while VaIN is often diagnosed incidentally, frequently in women with prior CIN or post-hysterectomy. Consequently, CIN is encountered far more often in clinical practice than VaIN.

While VaIN remains a relatively rare diagnosis compared to CIN, the broader impact of HPV vaccination programs may significantly reduce the burden of these less common but clinically significant conditions over time. Over the past decade, the introduction of prophylactic HPV vaccines has substantially altered the landscape of HPV-associated diseases. Clinical trials and real-world studies have consistently demonstrated that HPV vaccination significantly reduces the incidence of high-grade cervical intraepithelial neoplasia (CIN), and similar trends are now being observed for other HPV-mediated lesions, including VaIN, VHSIL, and AIN (Anal Intraepithelial Neoplasia [12,13].

Because VaIN is strongly linked to persistent infection with oncogenic HPV types, preventing initial infection through vaccination effectively lowers the risk of developing VaIN, particularly higher-grade lesions (VaIN 2/3), which carry a risk of progression to invasive vaginal carcinoma [3,20]. Evidence suggests that the greatest protective effect is achieved when the vaccine is administered before the onset of sexual activity, ideally during early adolescence [12]. Moreover, long-term follow-up studies indicate that the immunogenicity induced by the vaccine remains durable, supporting sustained protection against HPV infection and associated precancerous changes.

However, since the implementation of primary HPV testing and increased awareness among colposcopists, the incidence rates of VaIN are on the rise [21]. Particular attention to vaginal examination should be given to older patients with positive HPV screening tests [22] and those who have had a previous diagnosis of CIN or hysterectomy for HPV-related lesions [23].

### 4.1. Treatment Options

Treatment options are tailored according to disease extension, disease grading, patients’ characteristics, comorbidities, and previous treatments [24]. High-grade lesions have a preinvasive potential and should be treated even if there is no consensus on the optimal treatment. Regardless of the method, VaIN are at high risk of recurrence, with similar rates of recurrence observed with different treatments in our cohort as reported in the literature [6].

Ablative treatments should be carefully employed when abnormal epithelium is fully visualized and there is no concern of invasion, because a hidden lesion may not be reached by ablation and should be preceded by multiple mapping biopsies. Depth of destruction should be considered using ablative methods, since vaginal wall thickness modifies through time and reaches the lowest in postmenopausal women [25]. Thus topical estrogen should be used to improve thickness and to avoid damage to nearby structures during treatment. The cure rate of VaIN treated with ablation ranges between 73.5% and 86% [16], and in our series, it reached 86.28%, in line with better literature data.

On the other hand, excisional methods provide specimens for histopathological examination and help avoid missing invasive cancers. In our series, excisional treatment was associated with a higher rate of recurrence. This may be explained by multiple factors, including the multifocality of the lesion, the major risk factor for recurrence, and the lesion site. Almost half of VaIN cases are reported to be multifocal [1,26], and this may complicate both diagnosis and treatment.

Another possible explanation for the lower recurrence rate in the laser ablative arm might lie in the immune system stimulation of laser treatment, as many papers have addressed the immune boost of laser therapy to improve the uptake of viral antigen in host cells, thus enhancing antigen presentation [17,27]. A laser beam can modify the permeability of the cell membrane at the site of impact by a local thermal effect, and this allows a gene present in the surrounding medium to be transferred into the cell. This technology has been used in therapeutic HPV strategies to boost immune response, inducing specific CD4+ and CD8+ T cell responses and humoral immunity [28,29]

### 4.2. Risk of Progression

Our study confirms the risk of progression of high-grade VaIN after treatment, with 5/323 patients diagnosed with invasive carcinoma. Literature reports are between 2 and 12%, increasing with the duration of the follow-up [10]. The mean time to progression was 87.1 months in our series and 18 months in another large cohort [18]. Thus, it is mandatory to accurately explore the vagina during the diagnostic workup of CIN and cervical cancers and in oncological follow-up, given the chance that VaIN might be buried on the vaginal vault scar in case of positive vaginal margins.

We underline the significantly higher progression rate of VaIN3 compared to VaIN2, with all VaIN2 patients experiencing a VaIN3 diagnosis before progression. This may corroborate that VaIN3 is the real precursor of vaginal cancer, and it is the reason why pathologists should specify, if possible, the VaIN grade.

### 4.3. Limitations

The present study may have several limitations. The retrospective nature may limit results significance. Furthermore, results may not be comparable to general populations, as we considered patients referred to tertiary referral centers. Much demographic data were missing due to missing or old data, especially those risk factors linked to HPV-related lesions such as sexual practices, number of partners, and tobacco or alcohol use. Furthermore, HPV vaccination data are inconsistent due to lack of systematic vaccination and different vaccine types and schedules, and thus univariate and multivariate analysis was not feasible. However, the sample size is the second largest to date and the largest one from Europe, from two Italian referral centers for lower genital tract screening, adding significant information to the literature.

## 5. Conclusions

High-grade VaIN is an HPV-related preinvasive lesion whose risk of recurrence is higher when treated with excisional methods or in previously hysterectomized patients. Also, the risk of progression to invasive carcinoma after treatment is low and rises with time. Thus, long-term follow-up is needed to monitor the clinical outcomes and to avoid progression to invasion, especially in previously hysterectomized patients.

## Figures and Tables

**Table 1:** Baseline characteristics of the study cohort.

	All	VaIN 2 (*n* = 225)	Vain 3 (*n* = 98)	*p*-Value
**Center**				
Novara	50 (15.48)	38 (76%)	12 (24%)	0.2888
Torino	273 (84.52)	187 (68.5%)	86 (31.50%)	
**Age, years**				
Mean (SD, range)	55.43 (13.45, 24–93)	52.74 (12.37, 27–88)	61.59 (13.87, 24–93)	<0.0001
**Post-menopausal status**	159 (49.23%)	87 (54.72%)	72 (45.28%)	<0.0001
**Immunodepression (*n* = 317)**	24 (7.57%)	17 (70.83%)	7 (29.17%)	0.9289
**Site (*n* = 292)**				
Lateral walls	139 (47.60%)	111 (79.86%)	28 (20.14%)	<0.0001
Anterior/posterior walls	16 (5.48%)	11 (68.75%)	5 (31.25%)	
Vaginal vault	43 (14.73%)	15 (34.88%)	28 (65.12%)	
Fornix	94 (32.19%)	69 (73.40%)	25 (26.60%)	
**Treatment (*n* = 317)**				
Ablative	175 (55.21%)	142 (81.71%)	32 (18.29%)	<0.0001
Excisional	142 (44.79%)	81 (57.04%)	61 (42.96%)	
**Associated VHSIL**	17 (5.26%)	9 (52.94%)	8 (47.06%)	0.1234
**Associate Cervical HSIL**	112 (34.67%)	81 (72.32%)	31 (27.68%)	0.0165
**Vaccination Status**				
Primary vaccination	1 (100%)	1 (100%)	0 (0%)	0.8753
Vaccination before treatment	6 (1.86%)	3 (50.0%)	3 (50.0%)	
Adjuvant vaccination	42 (13.0%)	28 (66.67%)	14 (33.33%)	

ASCUS: atypical squamous cells of undetermined significance; ASC-H: atypical squamous cells cannot rule out high-grade squamous intraepithelial lesion; LSIL: low-grade squamous intraepithelial lesion; HSIL: high-grade squamous intraepithelial lesion; NILM: negative for intraepithelial lesion or neoplasia; VHSIL: Vulvar High-Grade Squamous Intraepithelial Lesion. Sites can be multiple; thus, percentages do not add up to 100%.

**Table 2:** Risk factors for recurrence.

			Univariable		Multivariable	
	No Recurrence (*n* = 252)	Recurrence (*n* = 71)	*p*-Value	HR Univariable [95% CI]	*p*-Value	HR Multivariable [95% CI]
**Center**						
Novara	40 (15.87)	10 (14.08)	0.8645	1.06 [0.54–2.07]	0.3517	1.48 [0.65–3.37]
Torino	212 (84.13)	61 (85.92)		ref		ref
**Age, years**						
Mean (SD)	54.09 (12.84)	60.17 (14.56)	0.0015	1.03 [1.01–1.05]	0.1175	1.02 [1.00–1.04]
**VaIN**						
2	183 (72.62)	42 (59.15)	0.0112	ref	0.8246	Ref
3	69 (27.38)	29 (40.85)		1.85 [1.15–2.97]		1.07 [0.58–1.97]
Hysterectomy	40 (15.87)	26 (36.62)	<0.0001	4.61 [2.41–8.84]	0.003	2.31 [1.09–4.90]
Cervical lesion	85 (34.98%)	27 (40.30)	<0.0001	2.60 [1.36–4.95]	0.068	1.96 [1.00–3.81]
Immunodepression	13 (5.26)	11 (15.71)	0.0056	2.49 [1.31–4.74]	0.1472	1.68 [0.83–3.37]
**Treatment**						
Ablative	151 (61.13)	24 (34.29)	<0.0001	ref	0.0013	ref
Excisional	96 (38.87)	46 (65.71)		3.28 [1.99–5.41]		2.87 [1.51–5.46]
**VHSIL**						
	8 (3.17)	9 (12.68)	0.0002	3.75 [1.85–7.59]	0.1946	1.69 [0.77–3.71]

**Table 3:** Clinical and histological characteristics of patients with VaIN2/3 that progressed to invasive cancer.

Age	Parity	Hysterectomy (Reason)	VaIN Grade at Initial Diagnosis	Recurrence	Time to Invasion	HPV Type	VaIN Site	Type of First Treatment
72	1	Yes (CIN3)	3	Yes	148.5	16	Vaginal vault	Excisional
59	0	Yes (pT1b cervical cancer)	3	Yes	15.7	16	Lateral walls III superior	Excisional
63	2	Yes (pT1a cervical cancer)	3	Yes	155.6	16	Lateral walls III superior	Excisional
43	0	No	2	Yes	28.0	Not available	Lateral walls III superior	Excisional
48	1	Yes (CIN3)	3	Yes	87.5	16	Vaginal vault	Excisional

## Data Availability

The data presented in this study are available on request from the corresponding author.
